# CRISPR/Cas9-editing of *PRNP* in Alpine goats

**DOI:** 10.1186/s13567-024-01444-1

**Published:** 2025-01-13

**Authors:** Aurélie Allais-Bonnet, Christophe Richard, Marjolaine André, Valérie Gelin, Marie-Christine Deloche, Aurore Lamadon, Gwendoline Morin, Béatrice Mandon-Pépin, Eugénie Canon, Dominique Thépot, Johann Laubier, Katayoun Moazami-Goudarzi, Ludivine Laffont, Olivier Dubois, Thierry Fassier, Patrice Congar, Olivier Lasserre, Tiphaine Aguirre-Lavin, Jean-Luc Vilotte, Eric Pailhoux

**Affiliations:** 1 Eliance, Paris, France; 2 UVSQ, INRAE, BREED, Université Paris-Saclay, 78350 Jouy-en-Josas, France; 3 BREED, École Nationale Vétérinaire d’Alfort, 94700 Maisons-Alfort, France; 4 INRAE, SAJ, 78350 Jouy-en-Josas, France; 5 INRAE, UE P3R Bourges, Domaine de Bourges, 31326 Osmoy, France; 6 INRAE, PAO, Nouzilly, France; 7 INRAE, AgroParisTech, GABI, Université Paris-Saclay, Jouy-en-Josas, France

**Keywords:** PRNP gene, PRION protein, goat, animal health, embryo editing, knockout

## Abstract

Misfolding of the cellular PrP (PrP^c^) protein causes prion disease, leading to neurodegenerative disorders in numerous mammalian species, including goats. A lack of PrP^c^ induces complete resistance to prion disease. The aim of this work was to engineer Alpine goats carrying knockout (KO) alleles of *PRNP*, the PrP^c^-encoding gene, using CRISPR/Cas9-ribonucleoproteins and single-stranded donor oligonucleotides. The targeted region preceded the *PRNP*^*Ter*^ mutation previously described in Norwegian goats. Genome editors were injected under the zona pellucida prior to the electroporation of 565 Alpine goat embryos/oocytes. A total of 122 two-cell-stage embryos were transferred to 46 hormonally synchronized recipient goats. Six of the goats remained pregnant and naturally gave birth to 10 offspring. Among the 10 newborns, eight founder animals carrying *PRNP* genome-edited alleles were obtained. Eight different mutated alleles were observed, including five inducing KO mutations. Three founders carried only genome-edited alleles and were phenotypically indistinguishable from their wild-type counterparts. Among them, one male carrying a one base pair insertion leading to a KO allele is currently used to rapidly extend a *PRNP*-KO line of Alpine goats for future characterization. In addition to KO alleles, a *PRNP*^*del6*^ genetic variant has been identified in one-third of founder animals. This new variant will be tested for its potential properties with respect to prion disease. Future studies will also evaluate the effects of genetic background on other characters associated with *PRNP* KO, as previously described in the Norwegian breed or other species.

## Introduction

The abnormal folding of the cellular PrP (PrP^c^) protein, a highly conserved protein in mammals, is at the root of prion diseases, a group of fatal neurodegenerative disorders affecting various species, including ruminants, deer and humans [1]. While many prion strains remain species specific, as their transmission does not cross the species barrier, others are zoonotic, such as the BSE strain (bovine spongiform encephalopathy) responsible for mad cow disease and the emergence of the human variant Creutzfeldt‒Jakob disease ([2, 3], for reviews). Genetically, susceptibility or resistance to prions is mainly determined by polymorphisms of the *PRNP* gene, which encodes PrP^c^ ([4], for review). Notably, *PRNP* alleles associated with lower susceptibility to prion diseases have been identified in sheep [5] and goats [6], enabling the significant reduction of classical scrapie incidence in these species by establishing rational mating practices to facilitate the propagation of these alleles within the flock [7]. Furthermore, full resistance to this pathology can be achieved by knocking out the *Prnp* gene in mice without affecting their survival under standard breeding conditions [8, 9]. This finding could be relevant in view of atypical scrapie strains and their possible involvement in the dissemination of BSE [10–12].

Recently, a KO allele of the *PRNP* gene was identified in Norwegian goats [13]. This allele, named *PRNP*^*Ter*^, confers resistance to scrapie in these animals [14] but is associated with metabolic peculiarities that might prove disabling under certain environmental conditions. These animals displayed myelination defects [15], which were also observed in some *Prnp*^*−/−*^ mouse lines [16]. This can induce neurological disorders in older animals. In addition, these goats exhibit alterations in their bone marrow physiology [17] and innate immunity [18, 19]. This observation may be correlated with the greater sensitivity of *Prnp*^*−/−*^ mice to viral infections, particularly influenza, which leads to higher mortality in infected mice of this genotype than in their wild-type counterparts [20, 21]. On the other hand, the potential appearance of abnormally folded PrP was observed in influenza-infected cultured neuroblastoma cells, suggesting a complex interaction between these two pathologies [22].

In *Prnp*^*−/−*^ mice, other studies have revealed certain behavioral abnormalities ([23–25], for example) that have not been studied to date, to our knowledge, in Norwegian goats. Interestingly, some of these phenotypes associated with *Prnp* gene KO in mice were found to depend on their genetic background [26, 27]. Indeed, although the biological function of PrP^c^ remains the subject of intensive study, its involvement in the regulation of oxidative stress ([28, 29], for example), neuroprotection [30, 31], development of some cancers ([32, 33], for reviews) and control of the immune response raises questions about the potential consequences of its invalidation during the onset of environmental stresses.

The advent of nuclease-based genome-editing techniques, particularly the CRISPR/Cas9 system, has led to applications in the search for animals resistant to pathologies. The *PRNP* gene was identified as a key target of these studies because of its major role in scrapie resistance [34, 35]. Indeed, the occurrence of new prion diseases in wild or semiwild species, such as chronic wasting disease in cervids and camel prion disease, raises fears of the potential emergence of new zoonotic strains [36–40] for which livestock might serve as intermediate hosts [41–43]. The acquisition of absolute genetic resistance to these pathogens would offer health security beyond that of the agricultural field.

In the present report, we describe the use of CRISPR/Cas9 to mimic the naturally occurring Norwegian goat *PRNP*^*Ter*^ mutation in Alpine goats. The obtained genome-edited goats will allow comparative assessment of the *PRNP*^*−/−*^-associated phenotypes under two distinct genetic backgrounds, which will in turn inform the choice of decision tree for implementing a strategy to fight these pathogens.

## Materials and methods

### Animals and ethics approval

The majority (*n* = 61) of the donor and recipient Alpine goats used in this study came from the INRAE experimental unit (Domaine de Galles, Avord, France). These animals were previously subjected to a genetic selection program, and their milk was used for the production of goat cheese (“Crottin de Chavignol”). All the donor and recipient goats involved were culled because of their inability to continue producing milk, which was associated with their age and/or mammary gland problems. Blood samples were drawn from these 61 goats, and AMH levels were assessed using a commercial ELISA kit (Anshlab®) to establish additional quantitative criteria for selecting donor goats (i.e., those with the highest anti-Müllerian hormone (AMH) levels). Eighteen other cull goats were obtained from the INRAE-AgroParisTech experimental unit of Grignon and were not tested for circulating AMH levels. All experiments were performed with the approval of the French Ministry for Higher Education, Research and Innovation, MESRI (accreditation numbers APAFIS#32242, #32248 and #31342), following the guidelines issued by two committees for ethics in animal experimentation (COMETHEA N°045 and CEEA VdL N°019). This project involving genome editing in farm mammals also received a favourable opinion from the INRAE Committee for New Breeding Techniques (NBTs). All scientists working directly with the animals possessed an animal experimentation licence delivered by French veterinary services. Three INRAE animal facilities participated in addition to those of Avord and Grignon, two experimental units, SAJ (Jouy-en-Josas) and PAO (Nouzilly), and one platform (CIMA—BREED unit—Jouy-en-Josas) dedicated to animal surgery. Donor and recipient culled goats received from Avord or Grignon were housed at SAJ for hormonal treatments; donors were euthanized at SAJ, embryo transfers were carried out at CIMA, and recipient goats were kept at SAJ until weaning of their young. Founder animals were then bred at PAO and at SAJ depending on their mutated allele.

### Hormonal treatment of donor goats for the production of one-cell embryos

**Figure 1:**
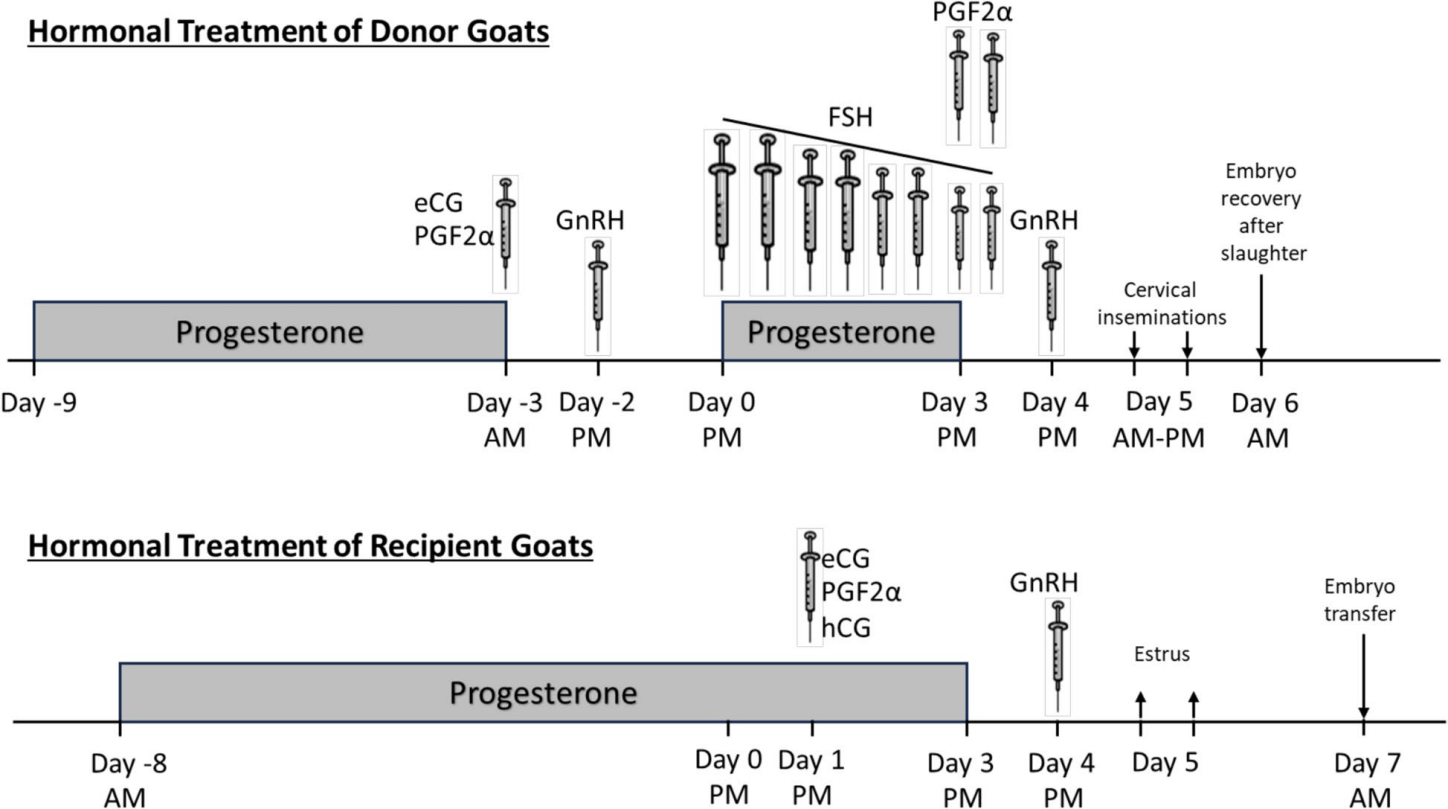
**Hormonal treatments of donor and recipient goats.**

The hormonal treatment of embryo donor goats followed a “Day 0 Protocol” adapted from previous studies [44–46] (Figure 1). The treatment started with the insertion of an intravaginal sponge (FGA: fluorogestone acetate, 45 mg) on day-9. On day-3, the intravaginal sponge was removed, and an intramuscular (IM) injection of eCG (equine ChorionicGonadotropin, Chronogest PMSG®, MSD Animal Health, 300 IU) and PGF2α (Prostaglandin F2 alpha or Estrumate®, 0.4 mL) was administered. Twenty-four hours later, an IM injection of GnRH (gonadotropin-releasing hormone = cystorelin®, 1 mL) was given. Two days later, theoretical ovulation occurred, marking day 0. The second phase of the treatment started with the insertion of a new intravaginal progesterone sponge (FGA: fluorogestone acetate, 45 mg). Concurrently, decreasing doses of FSH were administered (8 IM injections every 12 h of pFSH, 250 µg in total per goat with 2 × 47, 2 × 39, 2 × 23 and 2 × 16 µg (FSH; Reprobiol, Soiron-Pepinster, Belgium; one dose containing 500 µg of porcine FSH)). Two IM injections of prostaglandin (PGF2α) were given on the afternoon of day 3 and the morning of day 4. A final IM injection of GnRH (Cystorelin®) was administered on the afternoon of day 4. Two artificial cervical inseminations were performed on day 5 at 10:00 a.m. and 3:00 p.m. Uterine flushing was performed on day 6 after the donor goats were slaughtered. The genital tract was immediately removed and dissected to isolate and flush the oviducts with 20 mL of sterile Euroflush medium (IMV technologies, 019450). The ovulation points on the ovaries were counted, and then the embryos/oocytes were selected under a stereomicroscope.

### Single-cell embryo treatments with sgRNA/Cas9 RNPs and ssODNs

**Figure 2:**
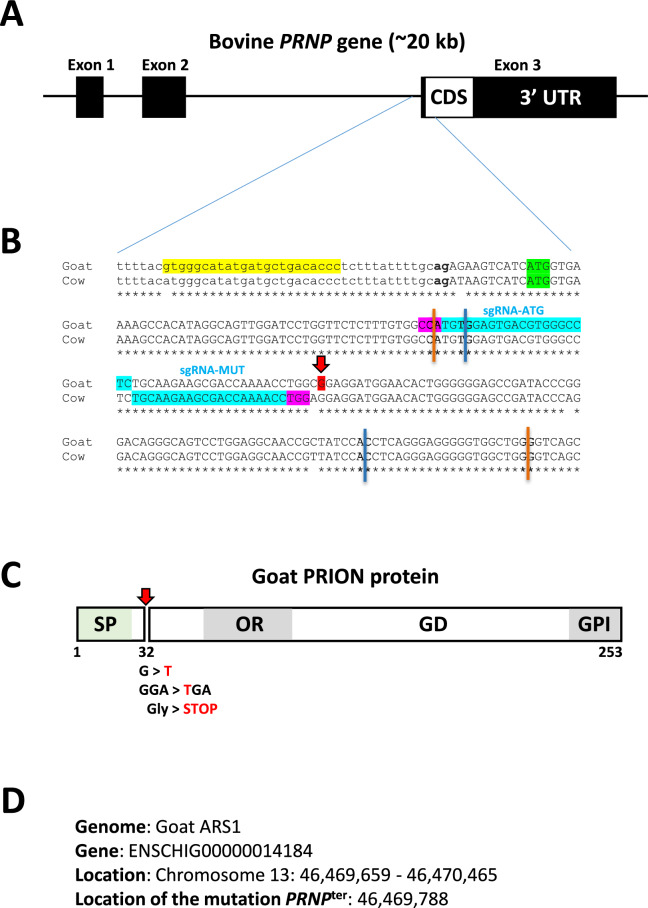
**Strategy for editing the Alpine goat PRNP gene.** **A** Schematic structure of the bovine *PRNP* gene. The coding sequence consists entirely of exon 3, as in goats. **B** Alignment of the targeted *PRNP* region in goats with that in their cow counterparts. The sequence in lowercase corresponds to the end of intron 2. The initiator codon ATG is highlighted in green. The Norwegian *PRNP*^*Ter*^ mutation is highlighted in red. The sequences highlighted in blue correspond to both sgRNAs, with the protospacer adjacent motif (PAM) in pink. The sgRNA-MUT is the sgRNA located closest to the Norwegian mutation and is indicated in the bovine sequence. The sequence in yellow corresponds to the primer PRNP3-F. A colored vertical line, orange for ssODN1 and blue for ssODN2, indicates the 5’ and 3’ ends of the ssODNs. **C** Scheme of the PrP protein showing the location of the stop codon due to the Norwegian mutation. SP: signal peptide; OR: octapeptide repeats; GD: globular domain; GPI: glycophosphatidylinositol anchor. **D** Precise location of the region in the goat reference genome.

The eggs recovered in vivo 18 h after the last cervical insemination, before DNA synthesis into 2-cell embryos [47], were denuded and injected under the zona pellucida with a complex containing the sgRNA located near the *PRNP*^*Ter*^ mutation designed using the CRISPOR software [48] (sgRNA-Mut, Integrated DNA Technologies (IDT), Figure 2), the TrueCut Cas9 v2 (Thermofischer) and one or the other single-stranded oligo-deoxynucleotide, ssODN (1/2/1, Alt-R HDR PS (4 phosphorothioate bonds)-modified, IDT, see also Table 1), by micromanipulation in embryo holding medium (EHM, Ref. 019449, IMV Technologies). After injection, presumptive zygotes were immediately electroporated using a NEPA21 type II Electroporator apparatus (NEPA GENE). This protocol combines micromanipulation, breaching the zona pellucida barrier, and electroporation of recently fertilized zygotes to deliver them with GE components, allowing excellent survival of embryos [49] while limiting their potential mosaicism [50]. Zygotes were cultured in 50 µL droplets of synthetic oviductal fluid (SOF) medium [51] and incubated at 38.5 °C in a humidified atmosphere containing 5% CO_2_ and 5% O_2_. These steps were performed on the morning of day 6 (Figure 1) and represented the starting point of embryo culture. Embryo cleavage was assessed 18 to 20 h later, and cleaved embryos were isolated and placed in straws at 35 °C until their transfer into recipient goats.

**Table 1:**
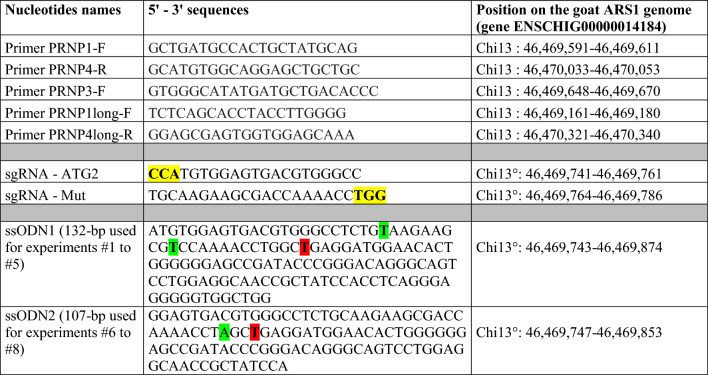
**Nucleotides used in the study**

The bases highlighted in yellow correspond to the PAM motif. The bases in green are silent mutations. The bases in red are the Norwegian mutations.

### Hormonal treatment of recipient goats

The day 0 treatment timeline for the recipient goats was the same as that for the donor goats (Figure 1). On day -8, an intravaginal sponge (FGA: fluorogestone acetate, 45 mg) was placed. On day 1, at 8 PM, the recipient goats received PGF2α (Prostaglandin F2 alpha or Estrumate®, 0.2 mL), eCG (equine chorionic gonadotropin, Chronogest PMSG®, MSD Animal Health, 300 IU), and human chorionic gonadotropin (hCG) (Chorulon®, 180 IU). On day 3, the intravaginal sponge was removed simultaneously with the sponge removal in the embryo donor goats. Finally, an IM injection of GnRH (Cystoreline®) was administered on the afternoon of day 4. The recipients were thus in oestrus at the same time as the embryo donors and therefore underwent embryo transfer 1 day after oestrus.

### Embryo transfer into recipient goats

Recipients fasted for 12 h prior to the surgical procedure. On the day of surgery, the goats received an intramuscular injection of an analgesic, butorphanol (Torbugesic®), at a dose of 0.04 mL/kg of body weight 20 min before induction. Anaesthesia was then induced by an intravenous injection of ketamine (Imalgene 1000®) at 0.04 mL/kg body weight and diazepam (Diazepam TVM5®) at 0.1 mL/kg body weight. The animal was placed supine on a heating pad throughout the procedure. After intubation, anaesthesia was maintained with inhaled isoflurane (1.5–4%), which was monitored and controlled via a respiratory assistance device. The abdomen was shaved and cleaned with Vetidine® soap and solution, and two incisions were made in the lower abdomen, 3 cm above the udder. These two incisions were used to introduce the laparoscopy tools (camera and forceps to immobilize the ovaries and verify ovulation in the recipient).

The ovary, where the ovulation point was observed, was exteriorized using the forceps. The infundibulum of the oviduct was then gently extended using two forceps. The embryos were introduced into the oviduct using a sterile micropipette. Once the transfer was completed, the incisions were sutured and covered with a protective and healing treatment, such as Aluspray®. The goats were then placed in an individual recovery box under surveillance until they awakened. Postoperatively, a systematic intramuscular injection of flunixin (Antalzen®) at 0.04 mL/kg was used to prevent pain.

### Genotyping of founder animals

Founder animals and their offspring were screened for the presence of mutations using genomic DNA extracted from ear clips. The *PRNP*-targeted region was amplified by PCR using primers PRNP1-F and PRNP4-R and the TaKaRa Ex-Taq enzyme (Takara Bio Europe). The PCR conditions included 40 amplification cycles: 94 °C-30 s, 60 °C-30 s and 72 °C-30 s. The 463-bp amplified fragment was Sanger sequenced by Eurofins Genomics (Courtaboeuf, France), with PRNP3-F used as an internal primer (Table 1). Mutations were deduced by comparing the obtained sequences with those of the *PRNP* goat ARS1 genome (Figure 2). In the case of mosaicism with different mutated alleles, the 463-bp PCR fragments were subsequently cloned and inserted into the pGEM-T easy (Promega) plasmid according to the manufacturer’s instructions, and the inserts from the twenty resulting recombinant plasmids were Sanger sequenced. To check for large insertion/deletion events, a 1.18 kb DNA fragment from the *PRNP*-targeted region was also amplified by PCR using the PRNP1long-F and PRNP4long-R primers and Sanger sequencing (Table 1). The following 40 amplification cycles were used: 94 °C for 30 s, 60 °C for 30 s and 72 °C for 60 s.

## Results

### Design of sgRNAs and homologous ssODNs for targeted recombination at the PRNP locus

The aim of this study was to reproduce the *PRNP*^*Ter*^ mutation described in the Norwegian goat population [13] into the Alpine genetic background. For this purpose, we designed and independently tested two single-strand RNA guides (sgRNAs), the sequences of which were chosen using the CRISPOR software [48], which target the Cas9 nuclease near the *PRNP*^*Ter*^ mutation site to induce a double-strand DNA cut 40 and 10 nucleotides upstream of the mutation site (Figure 2). To test their efficiency on in vitro-produced embryos derived from bovine ovaries that were collected at the slaughterhouse and reduce the number of donor goats following the 3R (refinement, reduction, and replacement) rules, two sgRNAs were designed in *PRNP* DNA regions that were fully homologous between goats and cows. This approach assumes that the relative efficiency of genome editing between the two guides observed in cattle will be similar to that in goats. Both sgRNAs were found to induce *PRNP* mutations in 90% of the injected bovine embryos (*n* = 60, data not shown). We thus decided to keep the guide closest to the *PRNP*^*Ter*^ mutated site (sgRNA-Mut) for the goat experiments (Figure 2). Furthermore, to reproduce the *PRNP*^*Ter*^ nonsense mutation (G>T conversion at position 46 469 788 on chromosome 13 of the goat ARS1 genome), by homologous recombination in the Alpine goat genome, we designed two ssODNs of 107 and 132 bp in length spanning the targeted region and carrying the *PRNP*^*Ter*^ base mutation (Figure 2).

### Goat zygote recovery and genome editing strategy

**Figure 3:**
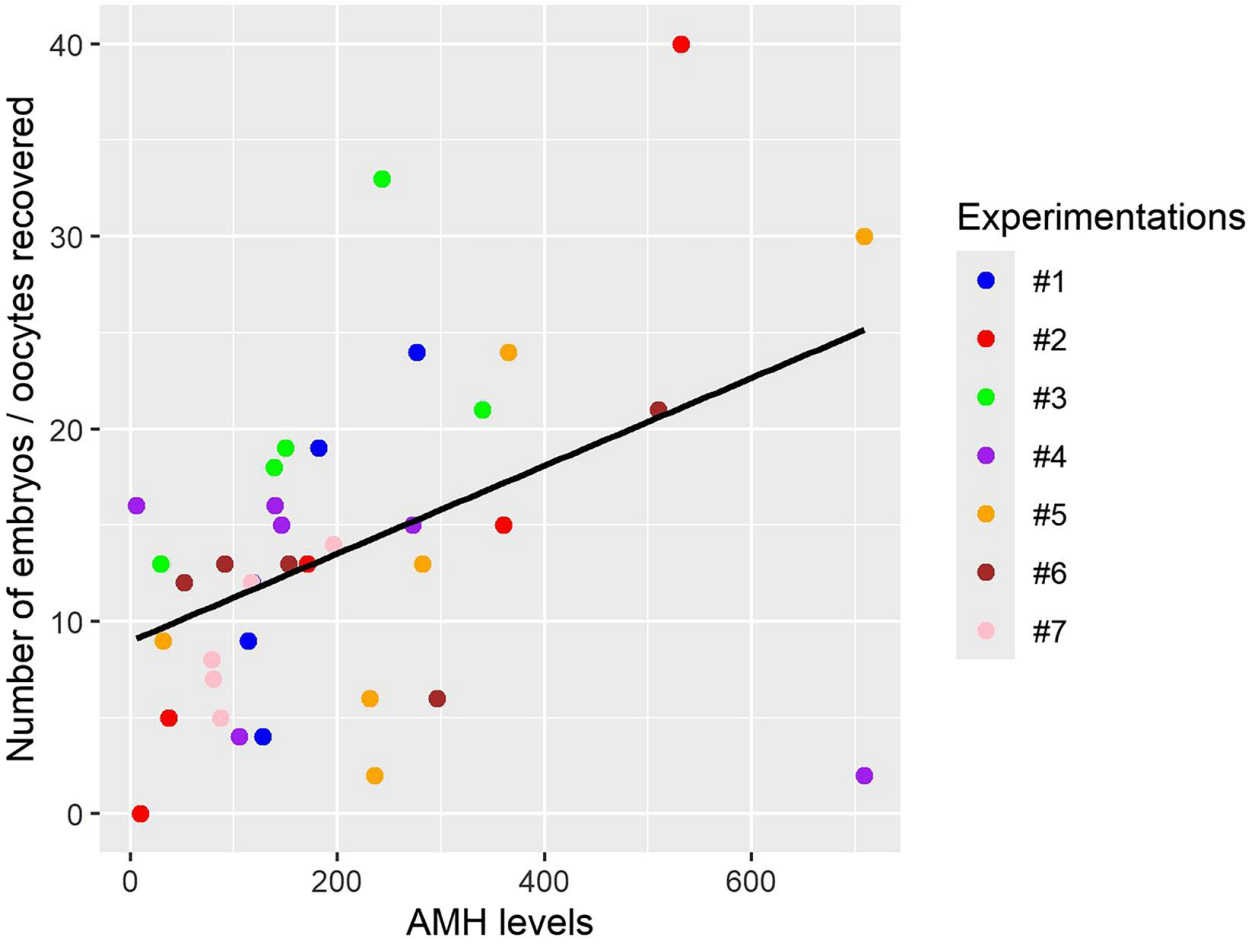
**Number of embryos/oocytes recovered in relation to the circulating AMH levels.** AMH levels were measured in the donor goats from experiments #1 to #7 and plotted on the x-axis. The number of embryos/oocytes recovered from each donor goat is plotted on the y-axis.

To comply with the 3Rs rule, we (i) carried out these experiments during the breeding season (autumn and early winter); (ii) when possible, we selected donor goats on the basis of their circulating AMH levels to optimize the number of collected and transferable embryos [52]; and (iii) used only cull animals for donor and recipient goats. The rationale behind the reform of these selected goats was primarily attributed to age-related or milking problems rather than fertility-related concerns. Overall, 41 embryo donor goats were used in eight experimental series, five of which were carried out at the end of 2021/beginning of 2022 and three during the following reproductive season. In total, 565 one-cell-stage embryos or oocytes were recovered, representing an average of 13.8 embryos per goat (Table 2). Selecting donor goats on the basis of the circulating AMH level for 7 of the 8 experiments performed might have contributed to this relatively high recovery rate, since we confirmed a correlation, although moderate (r = 0.4550807), between these two parameters (Figure 3). However, the observed coefficient of determination, r2 = 0.1844441, indicates that circulating AMH levels explain only 18% of the variance in the number of oocytes/zygotes obtained. The circulating AMH level is therefore a marker for predicting the ovulation rate in goats but is weak. Among the 565 embryos/oocytes manipulated for genome editing (GE), i.e., microinjection followed by electroporation (see Materials and methods), 526 survived this step and were cultured for ~ 20 h, ultimately producing 157 two-cell stage cleaved embryos (Table 2). This represents a cleavage rate of 29.3%, a rate likely reflecting the use of artificial insemination with frozen semen. In addition, the microinjection/electroporation process could have also reduced the cleavage rate, although this procedure was previously associated with excellent survival of embryos [49]. The embryos/oocytes remaining at the one-cell stage are either unfertilized oocytes or embryos blocked consecutively to the in vitro treatments. This uncertainty makes it difficult to assess the level of fertilization under our conditions.

**Table 2:** **Number of embryos/oocytes recovered from donor goats and treated for** **PRNP** **genome editing**

N° of experiment and date	Number of embryos/oocytes	Number of embryos/oocytes put in culture	Number of two-cells stage embryos	Cleavage rate (%)
#1—05/10/2021	68	67	23	34.3
#2—26/10/2021	73	67	18	26.9
#3—23/11/2021	104	98	38	38.8
#4—07/12/2021	68	62	21	33.9
#5—22/02/2022	84	72	19	26.4
#6—25/10/2022	65	65	7	10.7
#7—06/12/2022	46	45	6	13.3
#8—24/01/2023	57	50	25	50.0
Total	565	526	157	29.8

### Transfer of embryos to recipient goats and number of founder animals obtained

Following GE and overnight culture, embryos at the 2-cell stage were selected on the basis of gross morphology, with a particular focus on the appearance of a well-rounded and regular zona pellucida, and on the basis of two blastomeres of equivalent size that fully occupy the embryonic volume. In one experiment (#8), two embryos at the 4-cell stage were observed and selected. In the other experiments, the number of two-cell embryos transferred per goat ranged from 1–3, contingent on the number of two-cell embryos obtained. Among the 157 embryos obtained (Table 2), 122 were transferred to 46 recipient goats, 33 of which (72%) received three embryos, corresponding to the maximum number of transferred embryos per predetermined goat. (Table 3).

**Table 3:** **Number of transferred embryos and pregnancies**

N° of experiment and date	Number of recipient goats	Number of transferred embryos	Predicted pregnant goats at G21	Number of pregnant goats at G45	Pregnancy rate (%)	Number of newborns	Number of founders
#1—06/10/2021	6	18	1	0	0	0	0
#2—27/10/2021	6	18	2	0	0	0	0
#3—24/11/2021	6	18	5	3	50	6	4
#4—08/12/2021	6	18	2	0	0	0	0
#5—23/02/2022	7	16	2	0	0	0	0
#6—26/10/2022	4	5	0	0	0	0	0
#7—07/12/2022	5	11	1	0	0	0	0
#8—25/01/2023	6	18	5	3	50	4	4
Total	46	122	18	6	13	10	8

**Figure 4:**
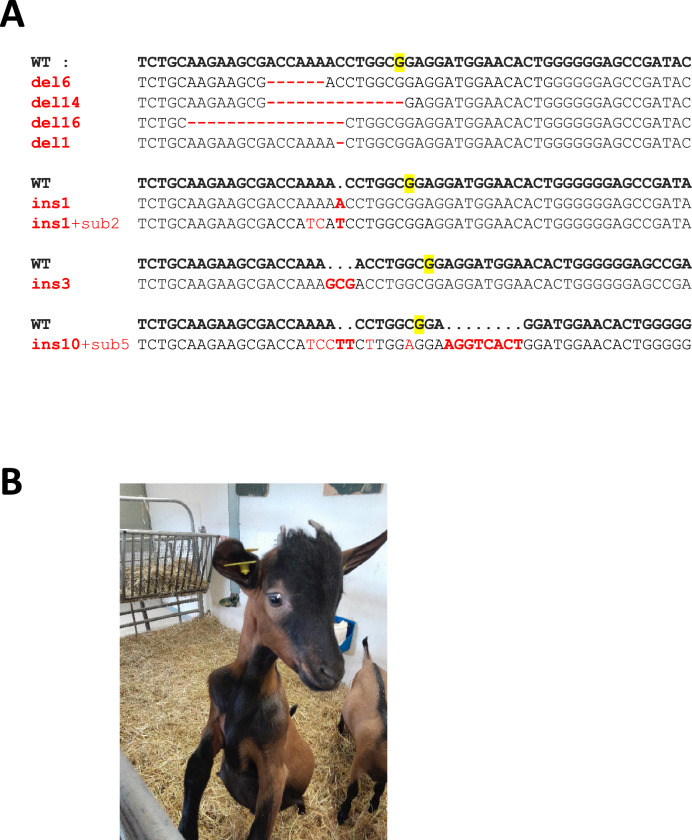
**Sequences of the eight mutated alleles generated and a picture of the César goat.** **A** The G highlighted in yellow corresponds to the Norwegian *PRNP*^*Ter*^ mutation at position 46,469,788 on chromosome 13 of the goat ARS1 genome. Deleted bases are replaced by a dash. Inserted bases are in bold. **B** Picture of César, the homozygous *PRNP*^*ins1/ins1*^ founder male.

The initial pregnancy diagnosis was performed at 21 days of gestation, G21 (day 0 representing the day of AI), by determining the circulating progesterone levels [53]. In all the series except one (#6), 1 to 5 recipient goats were predicted to be pregnant. Two series (#3 and #8) achieved high pregnancy rates, with 5 out of 6 goats (80%) testing positive (Table 3). Among the 18 positive goats at G21, only 6 were confirmed to be pregnant by echography at G45. These six goats naturally delivered one or two new-borns at term (G150 ± 3). In total, 10 newborns were obtained, comprising 5 males and 5 females (Table 4). *PRNP* genotyping by direct DNA sequencing of two different PCR amplicons was performed, revealing that eight out of these ten offspring carried at least one GE allele (Table 4 and Figure 4).

**Table 4:**
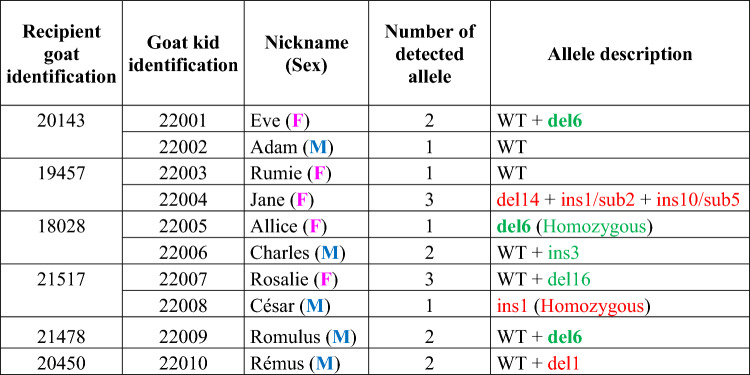
**Number of new-borns and founder goats**

The alleles in red are the predicted knockout alleles.

No founder animal carried the Norwegian *PRNP*^*Ter*^ mutation (Table 4 and Figure 4). This means that although DNA cuts were generated at the targeted *PRNP* locus and despite the presence of ssODN, no DNA repair by homologous recombination occurred. The twelve modified alleles detected in the eight founder animals originated from nonhomologous end-joining (NHEJ) processes (Table 4). One founder (designated Jane) was detected as mosaic with three genome-edited alleles. This founder goat already produced one F1 female offspring following breeding with a WT male. The transmitted mutation was *PRNP*^*ins10*+*sub5*^, one of those detected in the founder genome (Figure 4). Among the twelve detected GE alleles, the deletion of the same six base pairs (Figure 4) occurred 4 times independently. One female (Allice) was homozygous for this deletion (Table 4). This allele leads to a two amino acid deletion in the PrP protein. Crosses between founders of *PRNP*^*del6*^ are currently ongoing.

Finally, among the eight founder animals, two males, César and Rémus, were identified with a one-base pair insertion or deletion, respectively, which led to a frameshift allele and, consequently, a knockout allele (Table 4 and Figure 4). As César is homozygous for one base pair insertion (*PRNP*^*ins1/ins1*^), this male has been crossed with twenty WT Alpine goat females to extend this genetic variant and to rapidly derive Alpine goats with a KO at the *PRNP* locus.

## Discussion

The aim of the present study was to reproduce the KO observed in Norwegian goats at the *PRNP* locus in the Alpine breed by the use of GE. The methodology employed was based on the CRISPR/Cas9 system to target the homologous region mutated in the Norwegian breed. The objective was to reproduce this mutation in the Alpine breed through homologous recombination using an ssODN. The selection of gRNAs was carried out on bovine embryos obtained from slaughterhouse ovaries using the strong nucleotide homology that exists between goats and cows within the ORF of the *PRNP* gene. The selected gRNA was found to be approximately 95% effective in bovine embryos. Its transposition into a goat model allowed the birth of 10 offspring, 80% of which were GE. Thus, although these data remain limited, they strongly suggest high similarity in the effectiveness of GE between these two related species and validate our experimental approach, which aimed to minimize the number of animals used with respect to the 3Rs and to estimate the effectiveness of GE at preimplantation stages.

Circulating AMH concentrations have been identified as a predictor of the capacity of goats to produce high-quality embryos [52]. Furthermore, this criterion has been proposed to assess the ability of goats to respond to superovulation treatments [54]. Consequently, we used it to select the embryo donor goats, which was possible for 7 of the 8 experiments performed. Our data confirmed the correlation between the circulating AMH level and the number of embryos collected per goat. However, the low coefficient of determination obtained suggests that if this criterion is a potential indicator to consider, it should be associated with other yet unidentified factors to optimize this crucial step in GE experiments and to better align with the 3R criterion.

The proportion of offspring born represents 8.2% of the number of embryos reimplanted in recipient goats. This rate is lower than that reported by Niu et al. (30%) [55] or Zhou et al. (25.2%, with values ranging from 21.8 to 29.6%) [56]. In these previous experiments, embryos obtained by flushing donor goats underwent GE by microinjection of CRISPR/Cas9 RNAs. In our case, we combined microinjection and electroporation for GE, which could have weakened the development of the transferred embryos. Furthermore, in both of these studies, embryos were derived from natural mating after superovulation, whereas in our study, they were obtained through artificial insemination with frozen sperm. This experimental difference may also have contributed to the lower birth rate observed in our study.

In addition, a specific feature of the caprine species in comparison with the ovine species is the observation that, in the majority of the experiments (6 out of 8 in this study, representing 75%), no goats remain pregnant following embryo transfer. We observed the same phenomenon in two previous studies, although the approaches employed differed: the first used single-cell nuclear transfer and animal cloning [57], whereas the second used zinc-finger nuclease microinjection into one-cell embryos [58]. Notably, the two experiments involving pregnant goats (#3 and #8) presented the highest cleavage rates (38.8 and 50%, respectively), i.e., a parameter reflecting the time interval between fertilization and the first mitotic division of the embryo. This is not surprising since the timing of the first zygotic cleavage was demonstrated to be a marker of the developmental potential of mammalian embryos [59]. The remaining question is why the first zygotic cleavage is highly variable from one experiment to another in goats, despite the use of an identical protocol and the involvement of the same experimenters in all cases. Answering this question would also facilitate a reduction in the number of animals required for GE experiments. Nevertheless, our embryo selection criteria were entirely non-invasive and morphological. Several recent publications suggest that additional criteria can significantly increase the probability of success when associated with these morphological criteria. Among these are morphokinetic parameters [60–63], which include time-lapse measurements. Our observations, even if a limited number of embryos were obtained, also suggest that such non-invasive, morphokinetic criteria, associated with morphological criteria, would facilitate the optimization of pregnancy rates and, therefore, the number of GE animals obtained. Furthermore, this approach contributes to the 3R rule by limiting the number of recipients.

Despite the use of a protected ssODN (see Materials and Methods), no HR events were observed among the 10 different GE alleles. Even if the number of GE alleles remains limited, the absence of HR may appear surprising in view of the results obtained in the literature in cell culture or in vivo, including data on small ruminants ([64], for review). For example, despite a relatively low percentage of live offspring born alive carrying a GE allele (35%, 5 out of 17), Niu et al. obtained 71% of the GE alleles by microinjection of the defined point mutation (5 out of 7, [55]). In our experiment, we used two different ssODNs that followed the most current established rules for an optimized design [65, 66]. Briefly, the two ssODNs were (i) complementary to the sgRNA, (ii) asymmetric with arm lengths > 30 nt, (iii) the desired mutation was located proximally near the protospacer adjacent motif sequence, and (iv) they carried blocking mutations, avoiding recognition by the sgRNA of the modified sequence with either 2 mutations in the seed sequence or a mutation in the PAM sequence. These two ssODNs therefore presented a limited number of substitutions, a characteristic also supposed to improve HDR. A retrospective analysis of the sequence of the two ssODNs and, in particular, of the secondary structure potentially adopted by these single-strand DNAs via RNA folder software [67] could explain the absence of HDR. Indeed, these sequences exhibit a high proportion of self-folding, leading to the formation of a double-stranded structure (data not shown). This can inhibit the repair of double-strand breaks via synthesis-dependent strand annealing. Recently, it was also suggested that electroporation prior to the initiation of the S-Phase in goat embryos of a ribonucleoproteic complex (RNP) may increase the HR rate while reducing mosaicism [68]. As the exact reproduction of the Norwegian mutation at the *PRNP* locus was not a prerequisite for the success of this study, we did not attempt to use other ssODN sequences and/or to test different electroporation timings.

The observation that 33% of the GE alleles obtained by NHEJ presented the same 6-nucleotide deletion was unexpected. This deletion appears to preserve the reading frame of the *PRNP* gene ORF, potentially inducing the simple deletion of two amino acids in the N-terminal region of the mature protein. As previously indicated, the invalidation of the *PRNP* gene does not induce lethality. Therefore, the counterselection of nonsense mutations does not seem to be an explanation favouring the emergence of deletions preserving the ORF. One of the hypotheses considered would be that a secondary structure of the DNA in this region may favour the elimination of these 6 nucleotides by the nuclease. Despite the use of DNA structure prediction software [69], no such structure could be demonstrated (data not shown). This GE allele should lead to a caprine PrP protein lacking amino acids 29–30 (lysine (K) and proline (P)). This N-terminal region of the ovine PrP protein has been shown to play a role in the spread of certain prion strains in transgenic mice, although the implications of these two amino acids have not been precisely studied [70]. The properties conferred by this allele in goats in terms of sensitivity to scrapie and to other prion strains will therefore be particularly interesting to assess. To this end, the reproduction of goats carrying this allele is in progress, and their analysis will be an indirect result of this study.

The main objective of our study was to obtain null alleles at the *PRNP* locus in the Alpine breed that mimic or reproduce the natural mutation observed in the Norwegian breed. As previously discussed, identical mutations could not be reproduced. However, through NHEJ, five GE alleles are generated, inducing a frameshift and the introduction of a stop codon in the N-terminal portion of the protein, as observed in the Norwegian mutation. A goat carrying one of these alleles in a homozygous state was bred to quickly propagate this mutation and allow the rapid production of *PRNP*-knockout Alpine goats. The reproduction of the second goat, which is heterozygous for another allele, may be implemented to overcome a possible founder effect associated or not associated with an off-target mutation. If such an off-target mutation exists, it would influence the phenotype only if it was physically closely linked to the *PRNP* locus, as this would limit its segregation during reproduction. However, such an event is unlikely with respect to the selection criteria of the gRNA by CRISPOR [48].

The identification of Alpine goats devoid of PrP protein will make this possible, as announced in the expectations of the H2020 RUMIGEN program [71], to analyse the effects of genetic background on characters associated with this invalidation, such as those observed in the Norwegian breed [15, 17–19] or even in other species [16, 20, 21]. Furthermore, it allows the comparison of GEs with the classic genetic approach of introgression, which is currently underway, to transpose certain alleles from one breed to another without unduly affecting the character of the breed. The results of these studies will provide objective evidence of the potential benefits of a GE approach and enlighten public authorities on the classification of such animals, which are currently considered GMOs in Europe. These issues have also been the subject of a policy paper as part of the RUMIGEN project [72]. A significant outcome of this project will be the establishment of new *PRNP* genotypes in Alpine goats. The effects of these genotypes on resistance to prions and other traits of zootechnical interest will be evaluated by us and other members of the scientific community. Moreover, in the event of a change in European legislation, these animals could be made available to goat breeding companies. These animals are currently available to the scientific community and can be shared upon request.
